# Structural relationships among sportscasters’ speech components, online relationships, and viewing behavior

**DOI:** 10.3389/fpsyg.2026.1758758

**Published:** 2026-05-05

**Authors:** Yong-Seok Jang, Jae-Moon Lee, Sun-Young Lim

**Affiliations:** 1 Graduate School of Media and Communication, Kyung Hee University, Seoul, Republic of Korea; 2 Social Science Research Institute, Sungkonghoe University, Seoul, Republic of Korea; 3 Department of Physical Education, Seoul National University, Seoul, Republic of Korea

**Keywords:** online relationships, speech components, sportscasters, structural relationships, viewing behavior

## Abstract

This study examines the structural relationships among sportscasters’ speech components, online relationships, and viewing behavior in the context of sports media. Drawing on theories of parasocial interaction, source credibility, and uses and gratifications, this research conceptualizes speech as a multidimensional construct encompassing cognitive (logicality, verbal timing), affective (expressiveness), and perceptual (appearance, voice) elements. Data were collected from sports media users and analyzed using structural equation modeling to test the proposed hypotheses. The results indicate that all speech components significantly contribute to the formation of online relationships, confirming that both cognitive and affective communication cues play a critical role in fostering perceived intimacy with sportscasters. However, only affective and perceptual components—specifically expressiveness and appearance—exert direct effects on viewing behavior, while cognitive components influence behavior indirectly through online relationships. Furthermore, online relationship is found to have a significant positive effect on viewing behavior, demonstrating its mediating role in linking communication cues to audience engagement. These findings suggest that viewing behavior is primarily driven by emotional resonance and relational closeness rather than by informational clarity alone. This study extends the literature by providing an integrated framework that explains how multidimensional speech cues influence media consumption behavior through relational mechanisms. Practical implications highlight the importance of enhancing both expressive delivery and relational communication strategies in sports broadcasting.

## 1 Introduction

The rapid expansion of both professional and recreational sports into mainstream culture has been closely intertwined with the development of sports media. Media platforms not only disseminate sports-related information but also actively stimulate audience participation and consumption, thereby contributing to the growth and commercialization of the sports industry (Won and Ham, 2003; Pedersen et al., 2007). In this reciprocal relationship, sports organizations continuously supply diverse and high-quality content to media platforms, reinforcing a symbiotic ecosystem that has become increasingly complex in the era of digital and convergent media (Hutchins and Rowe, 2012; Jang, 2021).

In South Korea, a notable transformation in the sports broadcasting landscape has occurred with the shift from traditional terrestrial broadcasting to specialized sports channels. While terrestrial broadcasters have historically concentrated on mega-events such as the Olympic Games and the FIFA World Cup, the proliferation of sports-dedicated channels has enabled the diversification of content, including international leagues, niche sports, e-sports, and lifestyle-oriented programming (Kim, 2016; Boyle and Haynes, 2009). As a result, sports channels have emerged as key actors within the broader media ecosystem, playing a pivotal role in both content production and audience engagement (Lee et al., 2016).

To meet the increasing demand for diversified sports content, these channels produce a wide array of programming formats, including live broadcasts, highlight shows, news coverage, talk shows, and sports entertainment content (Jang, 2021). Central to these productions are sports announcers, including play-by-play commentators, field reporters, and studio hosts, who function as primary communicators between the media and audiences. Their roles extend beyond simple information delivery to include real-time narration, contextual interpretation, and emotional framing of sporting events (Bryant and Raney, 2000; Billings et al., 2015). As such, sports announcers represent a critical interface through which viewers experience and interpret sports content.

In an increasingly competitive and fragmented media environment, sports broadcasters seek to enhance viewer engagement and channel loyalty. Audience trust and repeated exposure are essential drivers of brand equity in media markets, particularly in the context of multichannel competition and platform convergence (Webster and Ksiazek, 2012; Napoli, 2011). Consequently, the communicative effectiveness of sports announcers has become a strategic asset, as their delivery style can significantly influence viewers’ perceptions, satisfaction, and continued engagement (Jang and Lee, 2021).

Previous research has established that communication competencies of media personnel are closely associated with audience outcomes, including satisfaction, credibility, and loyalty (Rubin and McHugh, 1987; Perse and Rubin, 1989). Broadcasters have also been evaluated based on attributes such as speech style, visual appearance, professional expertise, and demographic characteristics, all of which contribute to perceived program quality and credibility (Lee, 1998; McCroskey and Teven, 1999). From a theoretical perspective, mass communication involves the transmission of messages through mediated channels to large audiences, emphasizing both the content and the mode of delivery (McQuail, 2010).

Despite the recognized importance of announcers’ communication abilities, prior research has often operationalized speech in relatively narrow terms, focusing primarily on general verbal competence or isolated attributes. For example, Jang and Heo (2005) categorized expressiveness into verbal, vocal, and visual domains, which can be broadly grouped into verbal and nonverbal communication dimensions. This dichotomy aligns with foundational communication theories that distinguish between verbal and nonverbal modalities in shaping message effectiveness (Knapp et al., 2014; Burgoon et al., 2016). However, such binary classifications may overlook the multidimensional and integrative nature of communication cues in contemporary media contexts.

In particular, emerging research in media psychology suggests that communication effectiveness is best understood as a combination of cognitive, affective, and perceptual processes. Cognitive elements (e.g., logical structure and clarity) contribute to information processing and perceived expertise, while affective elements (e.g., expressiveness) facilitate emotional engagement. Perceptual factors (e.g., visual appearance and vocal qualities) further influence attention and impression formation (Lang, 2006; Nabi and Green, 2015). Nevertheless, empirical studies examining how these dimensions interact to influence audience behavior in sports media remain limited.

The evolution of digital media platforms—including social networking services (SNS), YouTube, and over-the-top (OTT) services—has further transformed the nature of audience–media relationships. Unlike traditional one-way broadcasting, contemporary media environments enable interactive and continuous engagement between viewers and media figures, fostering the development of online relationships that resemble interpersonal connections (Labrecque, 2014; Tukachinsky, 2011). These relationships are conceptually grounded in parasocial interaction theory, which posits that repeated mediated exposure can lead to perceived intimacy and relational bonds between audiences and media personalities (Horton and Wohl, 1956).

Moreover, the concept of social presence suggests that the perceived immediacy and intimacy of communication can enhance relational outcomes and engagement behaviors in mediated environments (Short et al., 1976). In sports media, where emotional intensity and real-time interaction are salient, the communicative style of announcers may play a crucial role in fostering such perceptions. Consequently, online relationships have emerged as a key mechanism linking communication cues to behavioral outcomes such as viewing intention, loyalty, and continued engagement (Dibble et al., 2016).

At the same time, the proliferation of media channels has led to increased audience fragmentation and selective exposure, making it more challenging for broadcasters to retain viewer attention (Webster, 2014). Although prior studies have examined sports media consumption and announcer characteristics (e.g., Kim and Lee, 2016; Seo et al., 2016), research specifically focusing on the role of announcers in specialized sports channels remains scarce. Furthermore, existing literature has often been limited in scope, focusing on specific subgroups (e.g., female announcers) or isolated attributes, rather than providing a comprehensive analysis of communication competencies across different announcer roles.

Given that sports announcers serve as the primary mediators between sports media and audiences, a systematic and multidimensional examination of their communication attributes is necessary. In particular, understanding how speech components influence both relational (online relationship) and behavioral (viewing behavior) outcomes can provide valuable insights into communication effectiveness in contemporary sports media environments.

Therefore, the purpose of this study is to investigate the effects of sportscasters’ speech components on viewers’ online relationships and viewing behavior. By integrating perspectives from communication theory and media psychology, this study aims to contribute to the literature by proposing and empirically testing a comprehensive framework that explains how multidimensional communication cues shape audience engagement in sports media contexts.

## 2 Theoretical background and hypothesis setting

### 2.1 Theoretical background: speech components and online relationship

In contemporary sports media environments, the formation of relationships between audiences and media figures can be effectively explained through the framework of parasocial interaction. Horton and Wohl (1956) conceptualized parasocial interaction as a phenomenon in which media users develop a sense of intimacy with media personas despite the absence of real, reciprocal interaction. Such relationships are strengthened through repeated exposure and can evolve into enduring psychological bonds. Subsequent studies have further expanded this concept, identifying parasocial interaction as a central mechanism underlying relationship formation in mediated environments (Dibble et al., 2016; Giles, 2002).

Within this context, sportscasters function not merely as conveyors of game-related information but as key communicative agents who facilitate emotional and social connections with audiences. Their speech characteristics serve as critical antecedents that shape viewers’ cognitive evaluations and emotional responses, which in turn contribute to the formation of online relationships.

First, informativeness and expertise represent fundamental determinants of trust formation. The perceived expertise and credibility of media figures have been identified as primary antecedents of parasocial relationship development, significantly influencing relational continuity and behavioral intentions (Dibble et al., 2016). In particular, studies conducted in social media and influencer contexts demonstrate that higher levels of information credibility and expertise lead to increased user engagement and interaction (Lou and Yuan, 2022). Accordingly, the sportscaster’s ability to deliver accurate and insightful commentary enhances viewers’ trust, which is likely to extend into the formation of online relationships.

Second, emotional expressiveness and delivery style play a pivotal role in fostering emotional engagement and relational closeness. Emotional expressions by media figures can evoke viewers’ affective responses and facilitate identification, thereby strengthening parasocial relationships (Kim et al., 2020a,2020b). In sports broadcasting contexts, vivid delivery and appropriate emotional expression enhance immersion in the game, leading to heightened emotional involvement. This emotional engagement has been shown to increase interactive behaviors in online environments, suggesting that expressive speech contributes positively to relationship formation through affective pathways.

Third, self-disclosure and perceived intimacy deepen the psychological bond between media figures and audiences. When media figures share personal experiences or emotions, viewers tend to perceive them as more authentic, which enhances liking and relational attachment. Nah (2022) found that self-disclosure positively influences message acceptance and parasocial relationship formation through perceived authenticity and liking. In the context of sports broadcasting, sportscasters who incorporate personal narratives or emotional reflections can reduce psychological distance, thereby facilitating stronger online relational ties.

Fourth, interactive speech cues serve as important mechanisms for enhancing parasocial interaction. The use of direct address or inclusive language (e.g., “we” or “together”) can create an illusion of real interaction, leading audiences to perceive mediated communication as socially engaging. Such perceived interactivity transforms media consumption into a social experience and has been shown to promote relationship formation and participatory behaviors (Hartmann, 2023). Therefore, the interactive elements embedded in sportscasters’ speech are likely to play a significant role in shaping online relationships.

In sum, sportscasters’ speech components contribute to the formation of online relationships through cognitive (expertise and trust), emotional (expressiveness and engagement), and social (interactivity and intimacy) dimensions. These multidimensional influences facilitate the development of parasocial relationships, which can subsequently extend into active online interactions. Accordingly, speech components can be conceptualized as key antecedent variables in the formation of online relationships within sports media contexts.

### 2.2 Theoretical background: speech components and viewing behavior

In contemporary sports media environments, viewing behavior extends beyond simple content consumption to encompass continued viewing intention, revisit intention, immersion, and online participatory behaviors. Such behaviors are influenced not only by the content itself but also by the characteristics of the communicator delivering the content. In this regard, sportscasters function as both information providers and experiential mediators, playing a crucial role in shaping viewers’ overall media experience. Accordingly, sportscasters’ speech components serve as key antecedents that evoke viewers’ cognitive and emotional responses, ultimately influencing their viewing behavior.

First, informativeness and expertise are critical determinants of viewers’ cognitive evaluations. In sports broadcasting contexts, the delivery of accurate and insightful information enhances viewers’ understanding and fosters trust in the content. This trust contributes to increased satisfaction, which in turn promotes sustained viewing behavior. Empirical evidence supports this relationship; for instance, Lou and Yuan (2022) demonstrated that the perceived expertise and credibility of media figures significantly influence users’ continued content consumption and engagement behaviors. This suggests that professional and informative speech by sportscasters plays a pivotal role in driving viewers’ behavioral responses.

Second, emotional expressiveness and delivery style are essential for enhancing emotional engagement. Sports content inherently involves emotional experiences, and the emotional expressions of sportscasters facilitate viewers’ affective responses and identification with the content. Dynamic commentary, heightened tension, and empathetic language can immerse viewers in the game, thereby increasing emotional involvement. Kim et al. (2020a,b) found that higher levels of emotional engagement and identification are associated with increased repeated viewing behavior, highlighting the role of emotional expression as a key mechanism linking communication style to behavioral outcomes.

Third, delivery style and vocal characteristics significantly influence the quality of the viewing experience. Elements such as articulation clarity, speech rate, intonation, and vocal tone enhance message effectiveness and facilitate both comprehension and immersion. These nonverbal cues contribute to the persuasiveness and attractiveness of communication, thereby shaping positive viewing experiences. Such experiences, in turn, lead to higher levels of revisit intention and channel loyalty, ultimately reinforcing viewing behavior.

Fourth, perceived intimacy and self-disclosure play a vital role in fostering emotional attachment. When media figures share personal experiences or emotions, viewers are more likely to perceive them as authentic, which enhances liking and relational attachment. Nah (2022) reported that self-disclosure positively influences message acceptance and behavioral intentions through perceived authenticity and liking. In sports broadcasting, sportscasters who present themselves in a relatable and humanized manner can strengthen viewers’ emotional bonds, thereby encouraging sustained viewing and participatory behaviors.

Furthermore, the effects of speech components on viewing behavior are closely associated with the process of parasocial relationship formation. Horton and Wohl (1956) proposed that repeated exposure to media figures leads to the development of perceived intimacy, which subsequently influences continued usage and loyalty behaviors. Later studies have consistently identified parasocial relationships as a key predictor of content consumption and engagement (Dibble et al., 2016; Giles, 2002). Thus, sportscasters’ speech components may influence viewing behavior both directly and indirectly through relational mechanisms.

In summary, sportscasters’ speech components—including informativeness and expertise, emotional expressiveness, delivery style, and perceived intimacy—operate across cognitive and emotional dimensions to shape viewers’ responses. These multidimensional influences ultimately contribute to the enhancement of viewing behavior, highlighting the critical role of communication characteristics in sports media contexts.

### 2.3 Theoretical background: online relationship and viewing behavior in sports media content

The rapid development of digital media environments has fundamentally transformed the way sports content is consumed. Audiences are no longer passive recipients; rather, they have become active participants who form relationships with media figures and content through online interactions. In this context, online relationships established during sports media consumption have emerged as a critical factor in explaining viewers’ behavioral responses.

Such online relationships are primarily grounded in the concept of parasocial relationships. Horton and Wohl (1956) described how media users develop psychological intimacy with media figures despite the absence of actual interaction. Subsequent research has emphasized that these relationships are closely linked to media consumption behaviors, extending beyond mere perception to influence concrete behavioral outcomes (Dibble et al., 2016; Giles, 2002).

Recent empirical studies have further demonstrated that parasocial relationships formed in online environments exert a direct influence on viewing behavior. For example, research on live-streaming content indicates that viewers’ parasocial relationships with media figures significantly enhance repeated viewing behavior and behavioral loyalty (Kim et al., 2020a,b). This suggests that stronger emotional bonds with specific media figures or content increase the likelihood of sustained engagement and continued consumption.

Moreover, online relationships extend their influence beyond passive viewing to a broader spectrum of behavioral outcomes. In media and communication contexts such as TV shopping, parasocial interaction has been found to significantly affect consumers’ behavioral intentions, including content usage and related engagement activities (Lu and Lu, 2022). These findings can be applied to sports media contexts, where relationships with sportscasters or commentators are likely to strengthen continued viewing and participatory behaviors.

Furthermore, online relationships play a crucial role in enhancing the qualitative dimensions of viewing behavior. Parasocial relationships formed during media consumption increase users’ immersion and engagement, which in turn lead to active participation such as commenting, content sharing, and fan-related activities. In second-screen environments, for instance, relationships with media figures have been shown to increase impulsive and participatory behaviors (Zhang et al., 2020). This indicates that online relationships function as a key mechanism that transforms viewing behavior from passive consumption into active engagement.

In addition, online relationships serve as an important mediating mechanism between attitudes and behaviors. Emotional engagement or identification with content does not directly translate into behavioral outcomes; rather, it is structured and reinforced through the formation of parasocial relationships, which subsequently lead to sustained viewing, loyalty, and content dissemination behaviors. This highlights the role of parasocial relationships as a critical mediating variable in explaining viewing behavior.

In summary, online relationships formed in the context of sports media content consumption significantly influence various dimensions of viewing behavior, including continued viewing intention, revisit behavior, participatory engagement, and loyalty. In particular, online relationships grounded in parasocial interaction strengthen viewers’ emotional bonds, thereby facilitating behavioral responses. As such, online relationship constitutes a key theoretical construct for explaining sports media consumption behavior.

### 2.4 Hypothesis setting and research model

**Figure 1:**
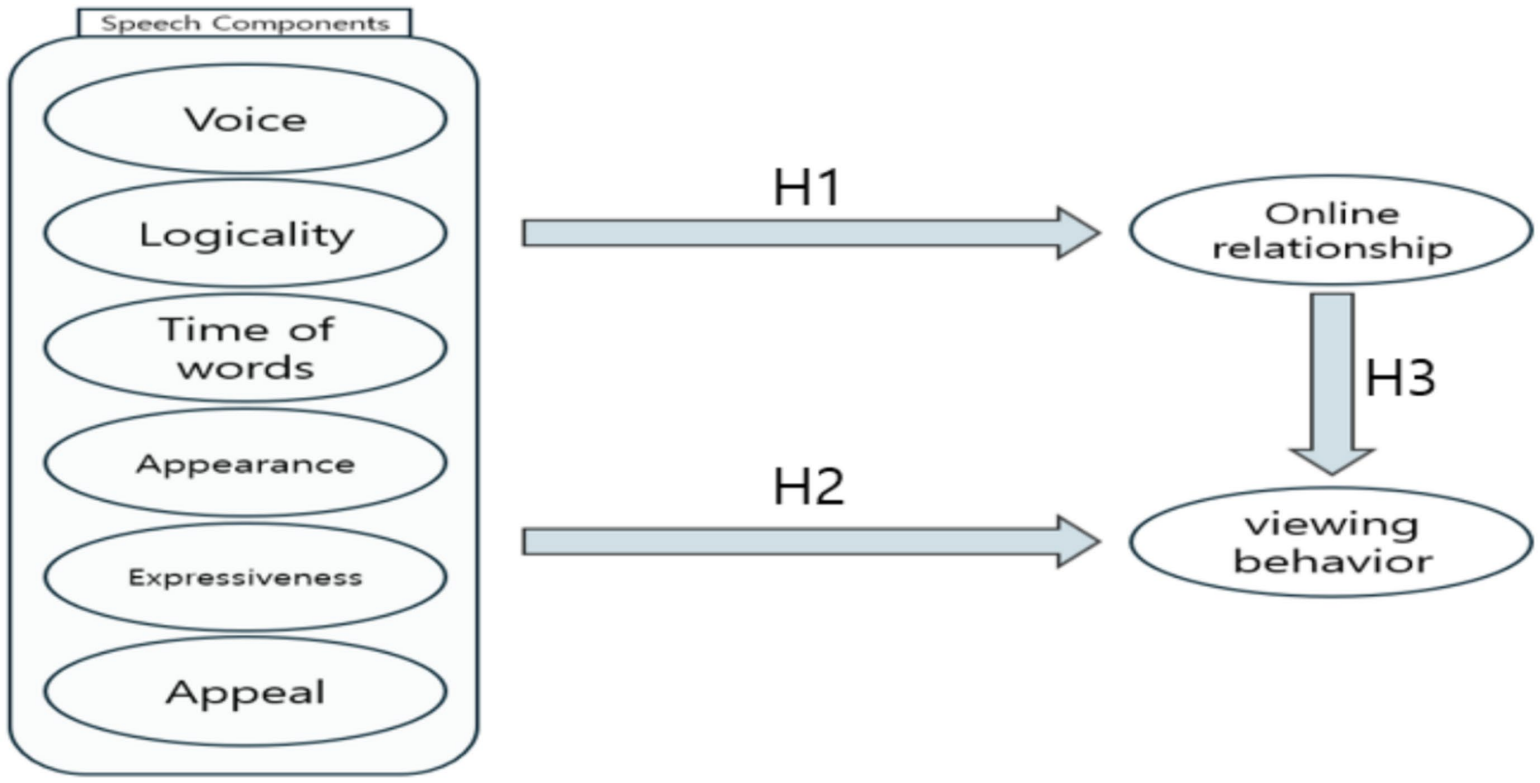
Research model.

We established the following hypotheses and models based on the results of previous studies (Figure 1):

*H1*. Sportscasters’ speech components have a significant effect on online relationship.

*H1-a*. Voice has a significant effect on online relationship.

*H1-b*. Logicality has a significant effect on online relationship.

*H1-c*. Time of Words has a significant effect on online relationship.

*H1-d*. Appearance has a significant effect on online relationship.

*H1-e*. Expressiveness has a significant effect on online relationship.

*H1-f*. Appeal has a significant effect on online relationship.

*H2*. Sportscasters’ speech components have a significant effect on viewing behavior.

*H2-a*. Voice has a significant effect on viewing behavior.

*H2-b*. Logicality has a significant effect on viewing behavior.

*H2-c*. Time of Words has a significant effect on viewing behavior.

*H2-d*. Appearance has a significant effect on viewing behavior.

*H2-e*. Expressiveness has a significant effect on viewing behavior.

*H2-f*. Appeal has a significant effect on viewing behavior.

*H3*. Online relationship in sports media content has a significant effect on viewing behavior.

## 3 Methods

### 3.1 Participants

To achieve the purpose of this study, data were collected from individuals with prior experience in viewing sports channel content. A non-probability sampling approach, specifically purposive sampling, was employed to recruit participants who met the inclusion criterion of having prior exposure to sports broadcasting content. This approach is considered appropriate for targeting information-rich cases relevant to the research objectives (Patton, 2015).

A total of 520 questionnaires were distributed, and all were returned, yielding a response rate of 100%. After data screening, six responses were excluded due to incomplete or inconsistent answers, resulting in a final sample of 514 valid cases for statistical analysis. The sample size exceeds the recommended threshold for structural equation modeling, ensuring adequate statistical power and model stability (Hair et al., 2019). The demographic characteristics of the participants are presented in Table 1.

**Table 1:** Demographic characteristics of participants.

Variables	Classification	Frequency (*n*)	Percentage (%)
Gender	Male	345	67.2
	Female	169	32.8
Age	10 s	65	12.6
	20 s	128	24.9
	30 s	116	22.5
	Over 40 s	105	20.4
Route of use	Smart phone	204	39.6
	PC	143	27.8
	TV	167	32.4
Total		514	100

Regarding ethical considerations, this study was conducted in accordance with established research ethics guidelines. As the study involved minimal risk, utilized anonymous survey data, and did not collect personally identifiable or sensitive information, it was classified as exempt from Institutional Review Board (IRB) review under applicable regulations. Participation was entirely voluntary, and informed consent was obtained from all respondents prior to data collection. Participants were assured of the confidentiality and anonymity of their responses, and all data were used solely for academic research purposes.

### 3.2 Measurement tool

This study was conducted to investigate the influence of sports channel announcers on online relationships and viewing behavior. The speech component questionnaire was used as a measurement tool based on previous studies by Jeffrey and Peterson (1980), Trenholm and Jensen (2000), Hickson et al., (2004), Jang and Heo (2005), and Kim (2006). Speech component factors were divided into verbal and nonverbal dimensions, and six speech component factors were modified and supplemented to fit the content of this study. The verbal dimensions were composed of phonetic factors (7 items), logical factors (6 items), sub-component factors (2 items), and timeliness factors (4 items), and the nonverbal dimensions of appearance (5 items) and expressiveness (3 items), for a total of 27 items. Online relationship and viewing behavior were modified and supplemented from items used in Levy and Windahl (1984), Son (2002), to fit the purpose of this study. The final questionnaire consisted of 39 items in total: 3 items on demographic characteristics of the research subjects, 27 items on speech components, 5 items on online relationship, and 4 items on viewing behavior. Each item was measured using a 5-point Likert scale.

### 3.3 Validity and reliability of measurement

Expert groups of professors (PhDs) in sports management verified the convergent validity of the measurement tools (questionnaires) used in this study. A confirmatory factor analysis (CFA) was performed to assess discriminant validity (Table 2).

**Table 2:** Confirmatory factor analysis.

Variables	Factors	SC	SE	*T*	C.R	AVE	Cronbach’s *α*
Voice	Tone of voice	0.904			0.934	0.670	0.950
	Voice intensity	0.892	0.073	13.11			
	Intonation	0.877	0.083	11.03			
	Vocal accent	0.847	0.024	9.32			
	Voice volume	0.836	0.065	10.11			
	Vocalization	0.832	0.032	13.22			
	Pronunciation	0.822	0.064	11.03			
Logicality	Logic	0.895			0.930	0.689	0.943
	Necessity	0.890	0.092	9.12			
	Message	0.867	0.089	11.33			
	Basis	0.861	0.078	12.24			
	example	0.854	0.054	9.12			
	Simplicity	0.834	0.051	12.43			
Time of words	Ready	0.877			0.912	0.722	0.895
	The first word	0.873	0.043	9.89			
	Context	0.872	0.067	10.12			
	Closing remarks	0.790	0.075	14.21			
Appearance	Appearance	0.886			0.914	0.686	0.855
	Hair style	0.811	0.034	14.23			
	body figure	0.810	0.054	16.23			
	Clothes	0.801	0.075	23.33			
	Makeup	0.541	0.076	4.32			
Expressiveness	Fac expression	0.889			0.874	0.698	0.877
	Gestures	0.876	0.043	15.43			
	Posture	0.851	0.087	16.43			
Appeal	Emotional	0.818		13.23	0.791	0.654	0.925
	Humor	0.781	0.038	9.12			
Online relationship	Friendliness	0.936			0.889	0.619	0.900
	Attractiveness	0.831	0.054	15.33			
	Familiarity	0.754	0.067	13.32			
	Relationality	0.741	0.056	15.32			
	Pleasure	0.704	0.034	0.17.32			
Viewing behavior	Watch it later	0.927			0.874	0.641	0.879
	Hope viewing	0.895	0.057	0.14.12			
	Recommendation	0.783	0.078	20.32			
	Experience	0.591	0.049	19.23			

*χ*^2^ = 311.236 (*df* = 113, *p* = 0.000), *Q* = 2.671, NFI = 0.852, TLI = 0.899, CFI = 0.892, RMR = 0.056, and RMSEA = 0.073.

The CFA analysis results were *χ*^2^ = 311.236 (*df* = 113, *p* = 0.000), Q value = (*χ*^2^/*df*) was 2.671, NFI = 0.852, TLI = 0.899, CFI = 0.892, RMR = 0.056, and RMSEA = 0.073. Since the *χ*^2^ value is determined by the value of [*F* × (sample size −1)], it is sensitive to the sample size, so even for the same model, rejection is determined by the sample size, making it difficult to properly evaluate the model itself. The Q value is used as an alternative to these problems; if the Q value is 3 or less, the model can be judged as suitable (Wheaton et al., 1977). Additionally, Bagozzi and Dholakia [48] evaluated that a model is suitable when NFI and CFI are 0.8 to 0.9 or higher, and RMR and RMSEA are 0.05 or 0.08 or lower. Accordingly, the results of the overall confirmatory factor analysis showed that these parts were relatively satisfactory, and overall, there was no problem in judging the fit index. The convergent validity of each variable, construct reliability (CR), and the average variance extracted (AVE) were calculated. The analysis revealed that the concept reliability of all variables was 0.587–0.749, and AVE was 0.850–0.980, satisfying the values of conceptual reliability (CR) of 0.7 or higher and AVE of 0.5 or higher as suggested by Hair et al. (2006), indicating that each variable demonstrates convergent validity. Next, to evaluate the reliability of the scale used in this study, an internal consistency reliability analysis method using Cronbach’s *α* coefficient was used, and the results of the analysis, using the theory of Nunnally and Bernstein (1994), revealed that the Cronbach’s *α* value must be 0.8 or higher.

## 4 Results

### 4.1 Correlation analysis

The results of analyzing the correlation between the speech components of sports announcers on sports channels and their online relationships and viewing behaviors are as shown in Table 3 above. All of the speech components, online relationships, and viewing behavior factors were found to have statistically significant correlations.

**Table 3:** Correlation analysis.

Variables	1	2	3	4	5	6	7	8
Voice	1							
Logicality	0.310	1						
Time of words	0.513	0.410	1					
Appearance	0.103	0.146	0.200	1				
Expressiveness	0.515	0.505	0.388	0.268	1			
Appeal	0.551	0.605	0.165	0.108	0.654	1		
Online relationship	0.364	0.378	0.377	0.035	0.432	0.297	1	
Viewing behavior	0.055	0.121	−0.008	0.250	0.024	0.138	0.116	1

1. Voice; 2. Logicality; 3. Time of words; 4. Appearance; 5. Expressiveness; 6. Appeal; 7. Online relationship; 8. Viewing behavior **p < 0.01.

### 4.2 Model verification

The results of the analysis verified the suitability of the structural model established in this study, which is as follows: *χ*^2^ = 311.236, *df* = 113, NFI = 0.832, TLI = 0.899, CFI = 0.892, RMR = 0.056, RMSEA = 0.073 (Table 4).

**Table 4:** Fit index of the research model.

A construct	*χ^2^*	*df*	*p*	NFI	TLI	CFI	RMR	RMSEA
Acceptance level	311.235	113	0.000	0.832	0.899	0.892	0.056	0.073
Acceptance criteria	—	—	—	More than 0.8 ~ 0.9	More than 0.8 ~ 0.9	More than 0.8 ~ 0.9	Less than 0.05 ~ 0.08	Less than 0.05 ~ 0.08

This is the same figure as the confirmatory factor analysis fit, because confirmatory factor analysis, wherein all relationships between latent variables are set as correlations, and structural equation modeling, wherein all relationships between latent variables are set as correlations or causal relationships, are equivalent models. Therefore, there were no statistical errors in determining the model fit indices for each variable.

### 4.3 Hypothesis testing

The structural equation model was used to confirm the causal relationship between the hypotheses established in this study and the research model variables (Table 5).

**Table 5:** Hypothesis testing result.

H	Path	SE	CR	*p*	Accept/reject
H1-a: voice → online relationships	0.153	0.070	2.348	0.019	Accept
H1-b: logicality → online relationships	0.214	0.068	0.3.362	0.000	Accept
H1-c: time of words → online relationships	0.135	0.055	2.173	0.000	Accept
H1-d: appearance → online relationships	0.107	0.051	2.289	0.000	Accept
H1-e: expressiveness → online relationships	0.304	0.060	4.672	0.000	Accept
H1-f: appeal → online relationships	−0.126	0.079	−1.638	0.103	Reject
H2-a: voice → viewing behavior	0.007	0.088	0.099	0.921	Reject
H2-b: logicality → viewing behavior	−0.094	0.085	−1.321	0.187	Reject
H2-c: time of words → viewing behavior	−0.049	0.069	−0.718	0.473	Reject
H2-d: appearance → viewing behavior	0.254	0.064	4.900	0.000	Accept
H2-e: expressiveness → viewing behavior	0.152	0.075	2.098	0.037	Accept
H2-f: appeal → viewing behavior	0.204	0.092	2.356	0.019	Accept
H3: online relationships → viewing behavior	0.116	0.058	2.253	0.025	Accept

***p < 0.001.

The structural model was analyzed to examine the causal relationships among sportscasters’ speech components, online relationship, and viewing behavior. The results are presented in Table 5.

First, the effects of speech components on online relationship were examined. The findings indicate that most sub-dimensions of speech components have a significant positive effect on online relationship. Specifically, Voice (H1-a) showed a significant positive effect (*β* = 0.153, *t* = 2.348, *p* < 0.05), as did Logicality (H1-b; *β* = 0.214, *t* = 3.362, *p* < 0.05), Time of Words (H1-c; *β* = 0.135, *t* = 2.713, *p* < 0.05), Appearance (H1-d; *β* = 0.107, *t* = 2.289, *p* < 0.05), and Expressiveness (H1-e; *β* = 0.304, *t* = 4.672, *p* < 0.05). Therefore, H1-a through H1-e were supported. In contrast, Appeal (H1-f) was not found to have a significant effect on online relationship (*β* = −0.126, *t* = −1.638, *p* > 0.05), leading to the rejection of H1-f.

Second, the direct effects of speech components on viewing behavior were assessed. The results reveal that only selected dimensions significantly influence viewing behavior. Appearance (H2-d) had a significant positive effect (*β* = 0.254, *t* = 4.900, *p* < 0.05), as did Expressiveness (H2-e; *β* = 0.152, *t* = 2.098, *p* < 0.05) and Appeal (H2-f; *β* = 0.116, *t* = 2.356, *p* < 0.05), supporting H2-d, H2-e, and H2-f. However, Voice (H2-a; *β* = 0.007, *t* = 0.099, *p* > 0.05), Logicality (H2-b; *β* = −0.094, *t* = −1.321, *p* > 0.05), and Time of Words (H2-c; *β* = −0.049, *t* = −0.718, *p* > 0.05) did not show significant effects on viewing behavior; thus, H2-a, H2-b, and H2-c were rejected.

Finally, the effect of online relationship on viewing behavior was tested. The results indicate that online relationship has a significant positive effect on viewing behavior (H3: *β* = 0.116, *t* = 2.253, *p* < 0.05), thereby supporting H3. Overall, the findings suggest that sportscasters’ speech components play a substantial role in shaping online relationships, while their direct effects on viewing behavior are limited to specific expressive and appearance-related factors. Furthermore, online relationship significantly influences viewing behavior, supporting its role as a key mediating variable within the proposed model.

## 5 Discussion

The present study aimed to examine the structural relationships among sportscasters’ speech components, online relationship, and viewing behavior, thereby providing both theoretical and practical insights into communication effectiveness in sports media contexts. Building upon the empirical findings, the following discussion is presented.

### 5.1 Effects of speech components on online relationship

The results demonstrate that multiple dimensions of sportscasters’ speech—voice, logicality, verbal timing, appearance, and expressiveness—significantly contribute to the formation of online relationships. This finding is consistent with the theoretical framework of parasocial interaction, originally proposed by Horton and Wohl (1956), which posits that repeated mediated exposure fosters perceived intimacy and relational bonds between audiences and media figures.

Subsequent research has reinforced this perspective by identifying communication cues as key antecedents of parasocial relationship formation (Dibble et al., 2016; Giles, 2002). In particular, the present findings extend prior literature by demonstrating that both cognitive (logicality, timing) and affective (expressiveness) dimensions of speech function as integrated drivers of relational development.

The significance of expressiveness aligns with emotional contagion theory (Hatfield et al., 1994), which suggests that individuals tend to synchronize emotions with expressive communicators. Similarly, the importance of logicality and informativeness supports the source credibility model (Hovland and Weiss, 1951), emphasizing that expertise and clarity enhance trust formation.

Furthermore, the effect of appearance corroborates findings from nonverbal communication research, which highlight the role of visual cues in shaping interpersonal evaluations and trust (Burgoon et al., 2016). Taken together, these results suggest that online relationship formation is a multidimensional process shaped by cognitive evaluation, emotional engagement, and visual perception.

### 5.2 Effects of speech components on viewing behavior

The findings indicate that appearance and expressiveness have significant direct effects on viewing behavior, whereas cognitively oriented components (e.g., logicality, voice clarity) do not exert direct influence. This suggests that behavioral engagement in media consumption is more strongly driven by affective and perceptual stimuli than by purely informational factors.

This result is consistent with uses and gratifications theory (Katz et al., 1973), which posits that media consumption is motivated by emotional and experiential needs. In sports media contexts, emotional arousal and enjoyment are particularly salient, making expressive delivery a critical determinant of viewer engagement.

Empirical studies further support this interpretation. For example, Kim et al. (2020a,b) found that emotional engagement significantly predicts repeated viewing behavior in live-streaming contexts. Additionally, research on media attractiveness suggests that visual appeal enhances attention, satisfaction, and behavioral intention (Reeves and Nass, 1996).

Thus, the present findings highlight that viewing behavior is primarily influenced by affective resonance and perceptual salience, rather than by cognitive processing alone.

### 5.3 Effects of online relationship on viewing behavior

The analysis confirms that online relationship significantly influences viewing behavior, supporting its role as a key mediating variable. This finding aligns with parasocial interaction theory, which suggests that perceived intimacy with media figures fosters loyalty and continued engagement (Horton and Wohl, 1956).

Recent studies have consistently demonstrated that parasocial relationships predict behavioral outcomes such as continued usage, loyalty, and participation (Dibble et al., 2016; Tukachinsky, 2011). In digital media environments, these relationships are further strengthened through interactive platforms, enabling more frequent and personalized engagement.

Moreover, the concept of social presence (Short et al., 1976) provides additional explanatory power. As viewers perceive a stronger sense of presence and connection with media figures, they are more likely to engage in sustained viewing behavior.

### 5.4 Integrated implications of speech components

This study contributes to the literature by conceptualizing speech components as multidimensional constructs that integrate verbal and nonverbal communication elements. The findings suggest that effective sports communication requires a holistic combination of cognitive clarity, emotional expressiveness, and visual appeal.

This interpretation is supported by classic communication research emphasizing the dominance of nonverbal cues in message interpretation (Mehrabian, 1971). Furthermore, media equation theory (Reeves and Nass, 1996) suggests that individuals respond to media figures as if they were real social actors, reinforcing the importance of integrated communication cues.

In conclusion, this study demonstrates that the utterance elements of sports commentators influence viewing behavior directly and indirectly through the formation of online relationships. Integrating communication theory and media psychology, the findings of this study provide a comprehensive framework for understanding viewer participation in the sports media environment.

## 6 Conclusion

This study provides a comprehensive examination of how sportscasters’ speech components shape viewing behavior through both direct and indirect mechanisms within contemporary sports media environments. By integrating perspectives from communication theory and media psychology, the findings offer several theoretically and practically meaningful conclusions.

First, the results confirm that sportscasters’ speech is not a unidimensional construct but a multidimensional communication system encompassing cognitive (e.g., logicality, timing), affective (e.g., expressiveness), and perceptual (e.g., appearance) elements. These dimensions jointly contribute to the formation of online relationships, supporting and extending parasocial interaction theory by demonstrating that relational bonds are simultaneously driven by informational credibility, emotional resonance, and visual cues. This reinforces the notion that audience–media relationships are constructed through an integrated processing of verbal and nonverbal signals rather than isolated communication factors.

Second, the study reveals a differentiated pathway through which speech components influence viewing behavior. While affective and perceptual elements (expressiveness and appearance) exert direct effects on behavioral engagement, cognitive elements primarily operate indirectly via online relationship formation. This finding advances existing literature by clarifying the mechanism through which communication effectiveness translates into actual media consumption behavior, suggesting that emotional and experiential gratifications are more immediate drivers of engagement, whereas cognitive evaluations contribute to longer-term relational attachment.

Third, the mediating role of online relationship underscores its central function as a psychological bridge between communication cues and behavioral outcomes. The results highlight that sustained viewing behavior is less a function of isolated content quality and more a consequence of perceived relational closeness and social presence. In increasingly interactive and personalized media environments, this relational dimension becomes a critical determinant of audience retention and loyalty.

From a practical standpoint, the findings suggest that sportscasters and sports media organizations should adopt a holistic communication strategy that simultaneously enhances clarity of information delivery, emotional expressiveness, and visual presentation. Training programs and performance evaluations should therefore move beyond traditional emphases on accuracy and expertise, incorporating affective communication skills and on-screen presence as core competencies.

Despite these contributions, several limitations warrant consideration. The cross-sectional design restricts causal inference, and future research should employ longitudinal or experimental approaches to validate the proposed relationships. Additionally, the study context may limit generalizability across different cultural or media environments, suggesting the need for cross-cultural validation. Further research could also explore moderating variables such as viewer characteristics, platform type, or genre of sports content to refine the explanatory model.

In conclusion, this study advances the understanding of communication effectiveness in sports media by demonstrating that sportscasters’ speech components influence viewing behavior through a complex interplay of cognitive, emotional, and relational processes. By highlighting the central role of online relationships, the findings provide a robust theoretical framework and actionable insights for enhancing audience engagement in the evolving digital sports media landscape.

## Data Availability

The original contributions presented in the study are included in the article/supplementary material, further inquiries can be directed to the corresponding authors.
